# Central nervous system mast cells in peripheral inflammatory nociception

**DOI:** 10.1186/1744-8069-7-42

**Published:** 2011-06-03

**Authors:** Dimitris N Xanthos, Simon Gaderer, Ruth Drdla, Erin Nuro, Anastasia Abramova, Wilfried Ellmeier, Jürgen Sandkühler

**Affiliations:** 1 Department of Neurophysiology, Center for Brain Research, Medical University of Vienna, Spitalgasse 4, 1090 Vienna, Austria; 2 Division of Immunobiology, Institute of Immunology, Center for Pathophysiology, Infectiology, and Immunology, Medical University of Vienna, Lazarettgasse 19, 1090 Vienna, Austria

**Keywords:** mast cell, dura, lumbar, capsaicin, carrageenan, cromolyn, spleen tyrosine kinase inhibitor

## Abstract

**Background:**

Functional aspects of mast cell-neuronal interactions remain poorly understood. Mast cell activation and degranulation can result in the release of powerful pro-inflammatory mediators such as histamine and cytokines. Cerebral dural mast cells have been proposed to modulate meningeal nociceptor activity and be involved in migraine pathophysiology. Little is known about the functional role of spinal cord dural mast cells. In this study, we examine their potential involvement in nociception and synaptic plasticity in superficial spinal dorsal horn. Changes of lower spinal cord dura mast cells and their contribution to hyperalgesia are examined in animal models of peripheral neurogenic and non-neurogenic inflammation.

**Results:**

Spinal application of supernatant from activated cultured mast cells induces significant mechanical hyperalgesia and long-term potentiation (LTP) at spinal synapses of C-fibers. Lumbar, thoracic and thalamic preparations are then examined for mast cell number and degranulation status after intraplantar capsaicin and carrageenan. Intradermal capsaicin induces a significant percent increase of lumbar dural mast cells at 3 hours post-administration. Peripheral carrageenan in female rats significantly increases mast cell density in the lumbar dura, but not in thoracic dura or thalamus. Intrathecal administration of the mast cell stabilizer sodium cromoglycate or the spleen tyrosine kinase (Syk) inhibitor BAY-613606 reduce the increased percent degranulation and degranulated cell density of lumbar dural mast cells after capsaicin and carrageenan respectively, without affecting hyperalgesia.

**Conclusion:**

The results suggest that lumbar dural mast cells may be sufficient but are not necessary for capsaicin or carrageenan-induced hyperalgesia.

## Background

Mast cells are primarily known for their role in Immunoglobulin E (IgE)-dependent hypersensitivity reactions, although their role in a wide range of physiological functions including neuroimmune interactions has been increasingly appreciated [1]. Activation of mast cells usually results in their degranulation, releasing preformed mediators, most notably histamine and chemokines while longer lasting activation results in the release of newly formed mediators such as cytokines, prostaglandins, and leukotrienes [2]. Mast cells are often found in proximity to sensory nerve endings and vasculature and their degranulation can modulate the excitability of nociceptive nerve endings [3]. They can themselves be activated by neurogenically-generated mediators such as substance P [4], various antigens, and inflammatory mediators released in pathophysiological states [5].

Peripheral mast cell activation is generally considered pro-inflammatory and pro-nociceptive. Inhibition of peripheral mast cells by chronic degranulation using intraplantar compound 48/80 blocks rodent capsaicin hyperalgesia [6]. Direct pharmacological inhibition by the often used mast cell "stabilizer" cromolyn also inhibits nociceptive behaviours in the second phase of the formalin test [7]. Cromolyn further inhibits neurogenic inflammation and edema induced by substance P or by low-frequency saphenous nerve electrical stimulation [8]. Multiple substances such as cyclooxygenase (COX) inhibitors [9] or spleen tyrosine kinase (Syk) inhibitors [10] are also known to inhibit mast cell activation and degranulation.

While a functional involvement of peripheral mast cells in inflammatory pain is well established, the role of central nervous system (CNS) mast cells is not well understood. Mast cells are not found in the spinal cord but mostly in the thalamus [11]. Significant sex differences and asymmetrical distribution which are estrogen-dependent are noted in thalamic mast cells in naive animals [12,13], and after nerve injury [14]. Mast cells have also been particularly investigated in the cerebral dura mater in relation to migraine pathophysiology and are thought to promote neurogenic inflammation and activation of meningeal nociceptors [15,16]. At the spinal level, dural mast cells are found at a significant density at the cervical, thoracic, and lumbar regions [17,18], although their functional roles have not been studied.

Since little to no white matter separates the lumbar dorsal horn from the subarachnoid and dura mater, it is hypothesized that mediators released from dura mast cells can reach the superficial laminae, a key relay station for nociception [19], to modulate synaptic transmission and nociception. While some of the individual mast cell mediators also produced by other immune cells are implicated in nociception and spinal synaptic transmission [20-22], the combination of mediators some of which serve anti-inflammatory and homeostatic functions [1], has not been specifically studied. To examine this, mast cells were cultured and activated *in vitro *and the mast cell supernatant was injected intrathecally to assess nociception. In addition, the supernatant was applied on the lumbar spinal cord to measure C-fiber evoked field potentials. Since intraplantar capsaicin induces spinal long-term potentiation (LTP) [23], a form of synaptic plasticity that amplifies nociception [19,23], we next examined spinal dural mast cell degranulation after intradermal capsaicin at various timepoints in awake conscious rats. We then examined whether intraplantar carrageenan, a model of non-neurogenic sterile peripheral inflammation, induces changes in number and degranulation of spinal dura and thalamic mast cells in male and female rats. Finally, we tested whether two intrathecally administered mast cell inhibitors, sodium cromoglycate (cromolyn) and the Syk inhibitor BAY-613606, are antinociceptive against capsaicin or carrageenan hyperalgesia.

## Results

### 1. Mast cell mediators can induce persistent nociception and long-term potentiation at spinal C-fiber synapses

**Figure 1:**
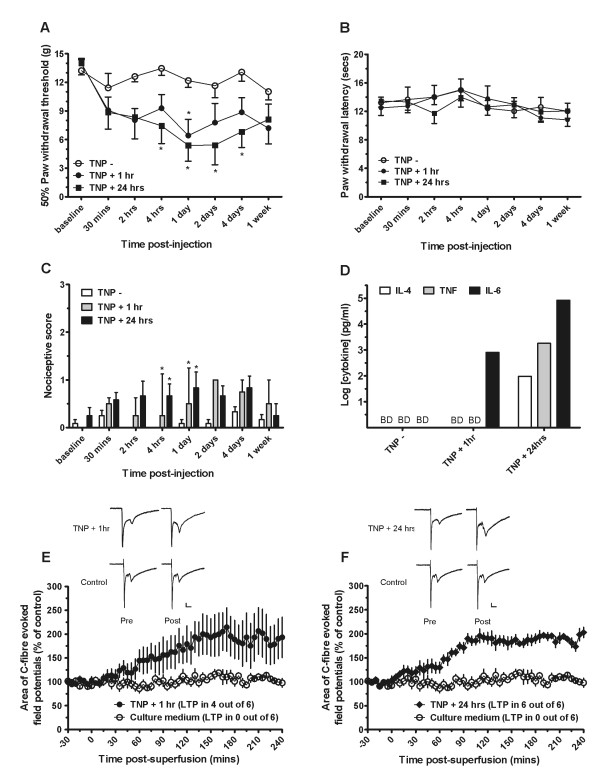
**Mast cell mediators are sufficient to induce persistent nociception and long-term potentiation**. Intrathecally injected supernatant from cultured mast cells activated for 1 hour induces significant mechanical hyperalgesia at 1 day post injection, while supernatant from mast cells activated for 24 hours induces significant mechanical hyperalgesia at 4 hours, 1 day, 2 days, and 4 days post-injection (* = P < 0.05) (A). No significant thermal hyperalgesia is seen (B), although cold allodynia is detected at 4 hours and 1 day post injection of supernatant from mast cells activated for both 1 hour and 24 hours (* = P < 0.05) (C). TNFα, IL-6, and IL-4 are detected in supernatant from 24 hour activated mast cells, and a lower level of IL-6 is detected in supernatant from 1 hour activated mast cells (BD: below detection). Experiment is representative from mast cell supernatant obtained from two independent batches of mast cells that were subsequently used in behavioral and electrophysiology experiments (D). LTP is elicited in 4 out of 6 animals from lumbar spinal application of supernatant of 1-hour stimulated mast cells (E), and in 6 out of 6 animals from lumbar spinal application of supernatant of 24-hour stimulated mast cells (F) (Calibration bars: 100 ms, 0.2 mV).

As shown in Figure 1A, intrathecal injection of mast cell supernatant from mast cells that were stimulated with either TNP (trinitrophenol) for 24 hours (TNP+24 hr) or 1 hour (TNP+1 hr) induced significant mechanical hyperalgesia at particular timepoints, as compared to supernatant stimulated with phosphate buffer saline (PBS) (TNP-). Two-way repeated measures analysis of variance (ANOVA) showed a significant effect of group (F_2, 105 _= 4.0, P < 0.05), time (F_7, 105 _= 11.08, P < 0.01) and interaction (F_14, 105 _= 2.35, P < 0.01). Post-hoc analysis revealed a significantly reduced mechanical threshold value at the 1 day timepoint of the 1 hour stimulated group as compared to control, but not at other timepoints. The TNP+24 hr group was significantly different from the TNP- group at 4 hours, 1 day, 2 days, and 4 days. None of the mast cell supernatants produced significant thermal hyperalgesia (Figure 1B). As for cold allodynia (Figure 1C), the Friedman test revealed a significant difference across the groups (Friedman statistic = 10.75, P < 0.01). Individual timepoint comparison with the Mann-Whitney test revealed significant differences between TNP- and TNP+1 hour at the 1 day (U = 8; P < 0.05) and 2 days (U = 4; P < 0.05) timepoints, as well as a significant difference between TNP- and TNP+24 hour at the 1 day (U = 7.5; P < 0.05) and 2 day (U = 5; P < 0.05) timepoints.

As shown in Figures 1E and 1F, supernatant from mast cells activated with TNP, but not application of cell culture medium, administered on the lumbar spinal cord induced spinal LTP in many animals. If an arbitrary criterion of a sustained (more than 2 hours) greater than 50% increase from baseline (over 30 minute intervals) is used to define LTP, it was assessed that none out of 6 control animals showed LTP, 4 out of 6 of 1-hour stimulated TNP animals, and all 6 of 24-hour stimulated TNP animals. Analysis of the averaged traces of C-fiber evoked field potentials showed no significant differences of the supernatant from mast cells activated for 1 hour with TNP as compared to control (Figure 1E). On the other hand, the supernatant from mast cells activated for 24 hours with TNP induced a highly significant increase in the area of C-fiber evoked field potentials as compared to control (Figure 1F) (P < 0.05). Area of A-fiber evoked field potentials was unchanged in all groups tested, as well as the paired pulse ratio, suggesting a post-synaptic effect (data not shown).

Mast cell supernatant used in behavioral and electrophysiological experiments was analyzed in order to confirm mast cell activation and the presence of newly formed cytokine mediators, tumor necrosis factor-α (TNFα), interleukin-4 (IL-4), and interleukin-6 (IL-6) (Figure 1D). While below detection in non-stimulated mast cell supernatant and in the 1-hour stimulated sample, TNFα was detected in the 24-hour stimulated sample at a concentration of 1819 pg/ml. Likewise, IL-4 levels were below detection in non-stimulated mast cell supernatant and in the 1-hour stimulated sample, while at 96 pg/ml in the 24-hour stimulated sample. IL-6 was also not detected in the non-stimulated mast cell supernatant, but was just above detection at 0.8 ng/ml in the 1-hour stimulated sample, and at a much higher concentration of 83 ng/ml in the 24-hour stimulated sample. We further tested some samples that had been primed but not stimulated with TNP versus those not primed and not stimulated with TNP and did not find any effect of priming on cytokine levels (data not shown). These results indicated that the 24 hour TNP mast cell activation was sufficient enough to induce the synthesis and release of newly formed cytokines.

### 2. C-fiber stimulation with capsaicin induces significant degranulation of lumbar dural mast cells

**Figure 2:**
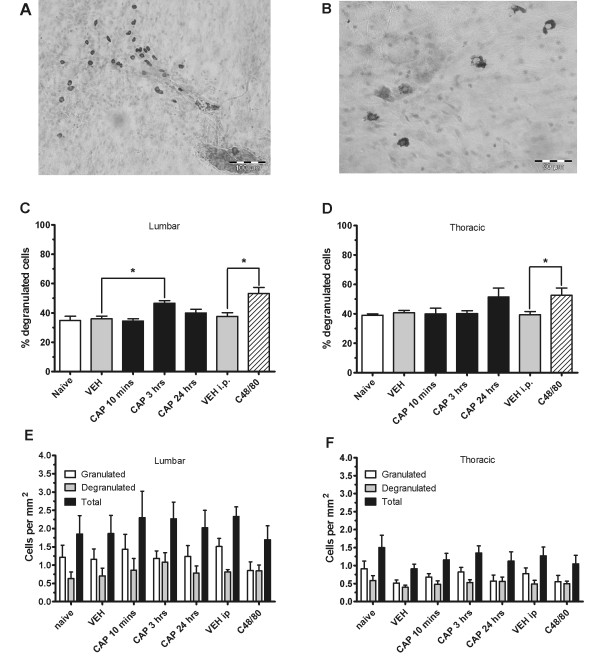
**Mast cell degranulation is increased 3 hours after capsaicin**. Mast cells are present in the lumbar dura and can be visualized with toluidine blue staining as mostly granulated with some being degranulated after vehicle treatment (A) or intensively degranulated after Compound 48/80 (B). C-fiber stimulation with capsaicin induces a significant increase in percent mast cell degranulation at 3 hours post-injection in the lumbar dura mater as compared to vehicle (C), but not in the thoracic dura mater (D) (* = P < 0.05). I.p. Compound 48/80 administration induces a significant increased in percent degranulation in both lumbar and thoracic dura mater (* = P < 0.05) (C, D). There are no significant changes in granulated, degranulated, or total mast cell density in neither the lumbar (E), nor the thoracic dura mater (F) after Compound 48/80 or capsaicin administration.

Lumbar and thoracic dura staining with toluidine blue revealed a significant number of mast cells both granulated and degranulated throughout the dura (Figure 2A). No definite anatomical pattern could be established, although there were sometimes particularly prominent clusters in the dorsal root entry zone. Degranulated mast cells could easily be identified by their loss of staining and ghostly appearance or by the scattering of individual granules (Figure 2B).

Animals treated with intradermal capsaicin were compared to vehicle at the 10 minute, 3 hour, and 24 hour timepoints (Figures 2C-F). As a positive control, an intraperitoneal injection of Compound 48/80 of 2 mg/kg which induces significant cranial mast cell degranulation [15] was used. This induced a significant increase in percent degranulation in the lumbar dura (t_12 _= 3.27; P < 0.01) (Figure 2C) and in the thoracic dura (t_12 _= 2.47; P < 0.05) (Figure 2D). One-way ANOVA for vehicle and capsaicin groups revealed a significant effect of group in percent mast cell degranulation in the lumbar dura (F_3, 27 _= 7.59; P < 0.01). Post-hoc analysis revealed a significant increase at 3 hours post-capsaicin as compared to vehicle (P < 0.01) (Figure 2C). There was no significant difference in the percentage of mast cell degranulation in the thoracic region after capsaicin (Figure 2D). ANOVA and t-tests showed that granulated, degranulated, and total cell density were not significantly different between vehicle and capsaicin groups, or between vehicle and Compound 48/80 administration in either the lumbar (Figure 2E) or the thoracic (Figure 2F) dura.

### 3. Inhibiting dural mast cell degranulation does not reduce capsaicin hyperalgesia

**Figure 3:**
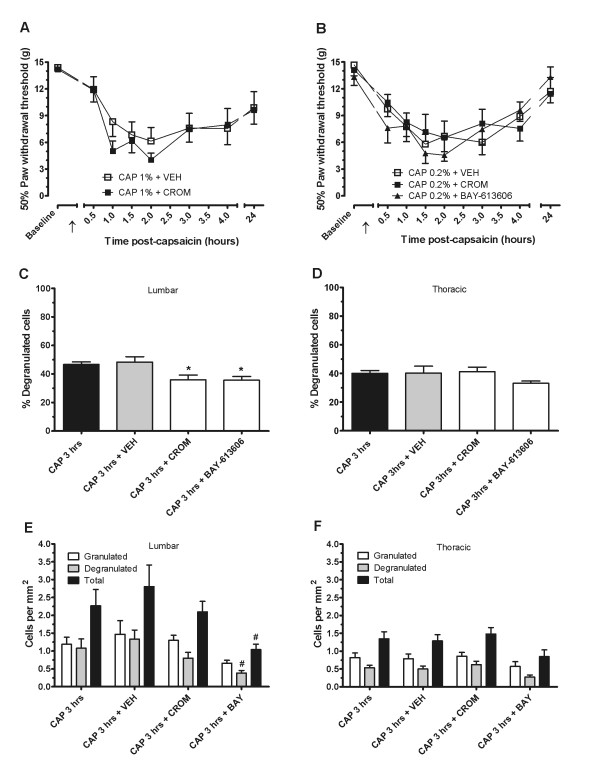
**Intrathecally administered mast cell inhibitors reduce mast cell degranulation but not capsaicin hyperalgesia**. Intrathecal cromolyn (200 μg) administered prior to intradermal 1% capsaicin (at timepoints shown by ↑) does not reduce mechanical hyperalgesia (A). There is also no effect of intrathecal cromolyn or BAY-613606 pretreatment on mechanical hyperalgesia (B) induced by 0.2% capsaicin. However, both BAY-613606 and cromolyn significantly reduce increased percent mast cell degranulation induced by 1% capsaicin in the lumbar dura mater (C) (* = P < 0.05 as compared to capsaicin alone), but not in the thoracic dura mater (D). BAY-613606, but not cromolyn, further reduces degranulated and total cell density in the lumbar dura mater (E) (# = P < 0.05 as compared to capsaicin + vehicle) but not in thoracic dura mater (F).

In order to test whether lumbar dural mast cells are necessary for capsaicin hyperalgesia, two mast cell inhibitors were used spinally to block lumbar dural mast cell degranulation. Intrathecal cromolyn pretreatment as compared to vehicle did not significantly reduce mechanical hyperalgesia induced by 1% capsaicin (Figure 3A). Repeated measures two-way ANOVA revealed a significant effect of time (F_7, 91 _= 11.83, P < 0.001), but no significant effect of group or interaction. Since 1% capsaicin may be confounded by stress effects particularly in the initial phase of flinching and primary hyperalgesia, we also tested a 0.2% capsaicin dose to better measure mechanical hyperalgesia and initial flinching behaviors. We did not detect significant reductions in flinching behaviors with either cromolyn or BAY-613606 pretreatment (data not shown). Further, pretreatment with cromolyn or BAY-613606 failed to reduce secondary mechanical hyperalgesia (Figure 3B). Repeated measures two-way ANOVA showed a significant effect of time (F_7, 147 _= 17.86, P < 0.001), but no significant effect of group or interaction.

In order to show that the mast cell blockers used in the behavioral experiments did indeed inhibit mast cells, different groups of animals were injected using the same administration protocol from behavioral experiments with cromolyn, BAY-613606, or vehicle prior to 1% capsaicin. One way-analysis revealed significant changes in percent mast cell degranulation between groups in the lumbar dura (Figure 3C; F_3, 27 _= 5.47; P < 0.01). Post-hoc analysis of the lumbar dura with the Dunnett's test showed that injection of vehicle did not significantly alter the percent degranulation, but this was decreased with cromolyn (P < 0.05) and BAY-613606 (P < 0.05), as compared to no drug. In the thoracic dura (Figure 3D), one-way ANOVA revealed no significant differences between groups. One-way ANOVAs of granulated, degranulated cell density after capsaicin, capsaicin + vehicle, capsaicin + cromolyn, and capsaicin + BAY-613606 revealed a significant effect of group for total mast cell density (F_3, 27 _= 3.21; P < 0.05) and degranulated mast cell density (F_3, 27 _= 3.21; P < 0.05) in the lumbar region (Figure 3E). Post-hoc analysis with Dunnett's test revealed a significant difference for BAY-613606 as compared to capsaicin + vehicle for both total cell density (P < 0.05) and degranulated cell density (P < 0.05). A significant difference in degranulated mast cell density was also detected by ANOVA in the thoracic region (F_3, 27 _= 3.21 P < 0.05), although neither individual comparisons to capsaicin alone nor to capsaicin + vehicle were found to be significant (Figure 3F).

### 4. Longer lasting peripheral inflammation with intradermal carrageenan induces increased lumbar mast cell density in female rats but not in male rats

**Figure 4:**
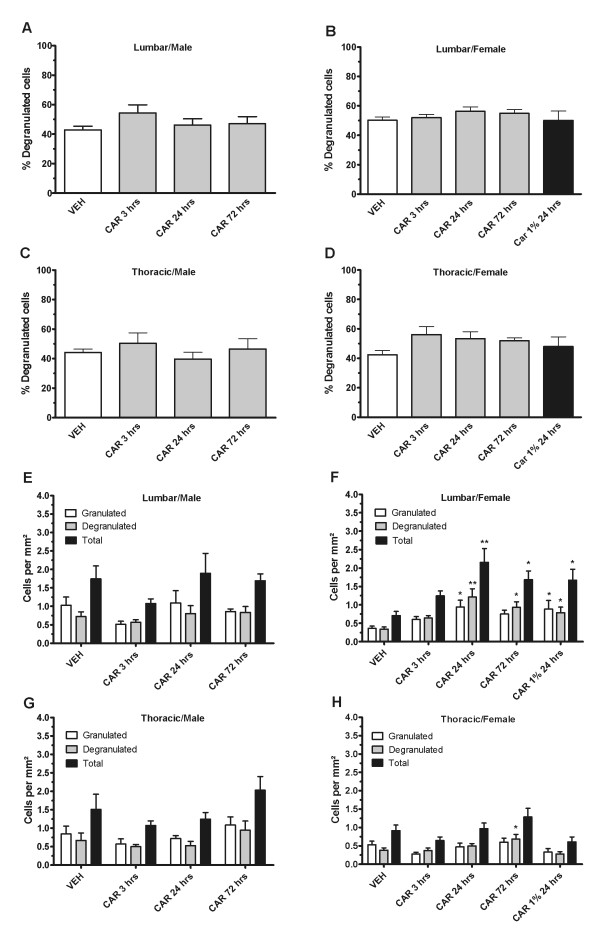
**Mast cell density is increased in the lumbar dura mater at 24 hours and 72 hours post-carrageenan in female rats**. Intraplantar carrageenan does not induce a significant increase in percent degranulation or any changes in total, granulated, or degranulated cell densities in male rats in the lumbar (A, E) or in thoracic (B, G) dura mater. There is no significant increase in percent mast cell degranulation after carrageenan treatment in female rats in the lumbar dura mater (B) or in the thoracic dura mater (D). However, intraplantar carrageenan induces a significant increase in total, granulated and degranulated mast cell densities in the lumbar dura mater at 24 hours post-injection and in degranulated and total mast cell densities at 72 hours post-injection (* = P < 0.05, ** = P < 0.001) (F). 1% carrageenan also induces increased total, granulated and degranulated mast cell densities at 24 hours post-injection in the lumbar dura mater (F). Degranulated cell density is increased in the thoracic dura mater of female rats at 72 hours post-injection (H).

Lumbar and thoracic dural mast cells were quantified after intraplantar carrageenan in male and female rats. Unlike capsaicin, in male rats, ANOVA showed no significant differences in percent degranulation in either the lumbar (Figure 4A) or thoracic (Figure 4C) dura mater. ANOVA also showed no significant effect of group for total, granulated, or degranulated mast cell density in lumbar (Figure 4E) or thoracic dura (Figure 4G). Since there are several reports of sex differences for mast cells in the literature [3,12,14], we also tested female rats. Here, again, we did not find any significant differences between groups for percent degranulation in neither the lumbar dura mater (Figure 4B) nor the thoracic dura mater (Figure 4D). However, when examining cell densities in the lumbar dura mater (Figure 4F), we found a highly significant effect of group for total mast cell density (F_4, 40 _= 6.96; P < 0.001), granulated mast cell density (F_4, 40 _= 3.81; P < 0.05), and degranulated mast cell density (F_4, 40 _= 7.61; P < 0.001). Post-hoc analysis revealed that in comparison to vehicle, there was a highly significant increase in total lumbar mast cell density and degranulated mast cell density at 24 hours post-carrageenan (P < 0.001) and at 72 hours post-carrageenan (P < 0.01). We also included a dose of 1% carrageenan in this analysis which also showed increased lumbar degranulated mast cell density at 24 hours post-carrageenan (P < 0.05). Granulated cell density was significantly different from vehicle in the 24 hour post-carrageenan group (P < 0.01) and in the 24 hour 1% post-carrageenan group (P < 0.05). In the thoracic dura mater (Figure 4H), ANOVA revealed a significant effect of group for thoracic degranulated mast cell density (F_4,40 _= 3.32; P < 0.05), but not of granulated or total mast cell density. Post-hoc analysis revealed a significant increase in degranulated cell density only at the 72 hours post-carrageenan timepoint as compared to vehicle (P < 0.05).

### 5. Thalamic mast cells are not significantly altered after 3% carrageenan

**Figure 5:**
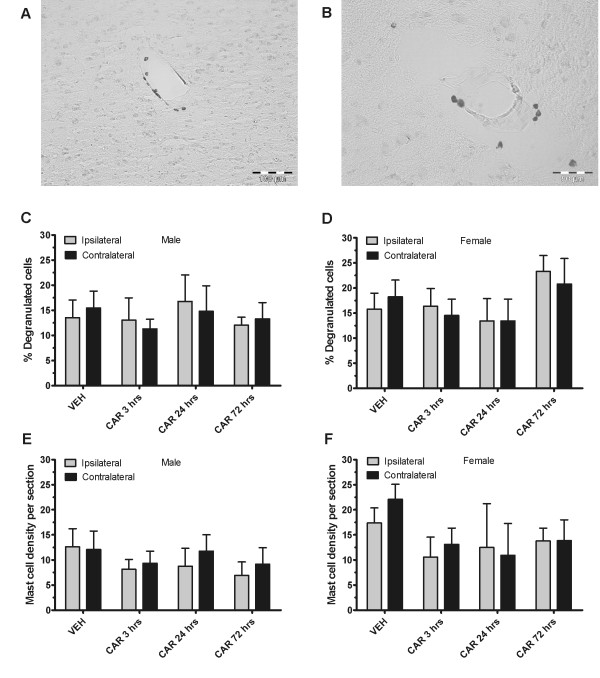
**Thalamic mast cell degranulation or density is not changed after carrageenan in neither male nor female rats**. There are significant numbers of mast cells in the posterior thalamus which are usually close to blood vessels (examples in A and B). Male and female carrageenan-treated rats do not show differences in percent mast cell degranulation as compared to vehicle, and there are also no significant ipsilateral/contralateral hemisphere differences in percent mast cell degranulation (C, D). Average mast cell number per section is also not significantly different between carrageenan treatments and vehicle in male and female rats, and there are also no significant ipsilateral/contralateral hemisphere differences (E, F).

Mast cells of the posterior caudal half of the thalamus were quantified from the same animals that were used for the dural preparation. Mast cells were easily visible mostly near blood vessels after toluidine blue staining (Figures 5A and 5B) and were quantified and assessed for degranulation status, both on the ipsilateral and on the contralateral sides of 18-20 non-consecutive thalamic slices (every second slice was successfully collected). ANOVA did not reveal significant differences in percent degranulation after carrageenan and vehicle treatments neither in the ipsilateral, nor in the contralateral thalamic hemisphere in male (Figure 5C) and female rats (Figure 5D). Likewise, there were no significant differences in mast cell density after carrageenan and vehicle treatments in male (Figure 5E), or in female rats (Figure 5F). This result suggests that increased spinal dural mast cell density after carrageenan treatment in female rats is a localized effect. We also investigated the estrous cycle stage at the time of animal sacrifice and found that it was evenly distributed in vehicle treated groups. Our small sample sizes over the different estrous groups, as well as the conditions in the current study did not allow statistically relevant correlational studies with mast cell degranulation.

### 6. Inhibiting increased degranulated spinal dural mast cell density does not reduce carrageenan hyperalgesia

**Figure 6:**
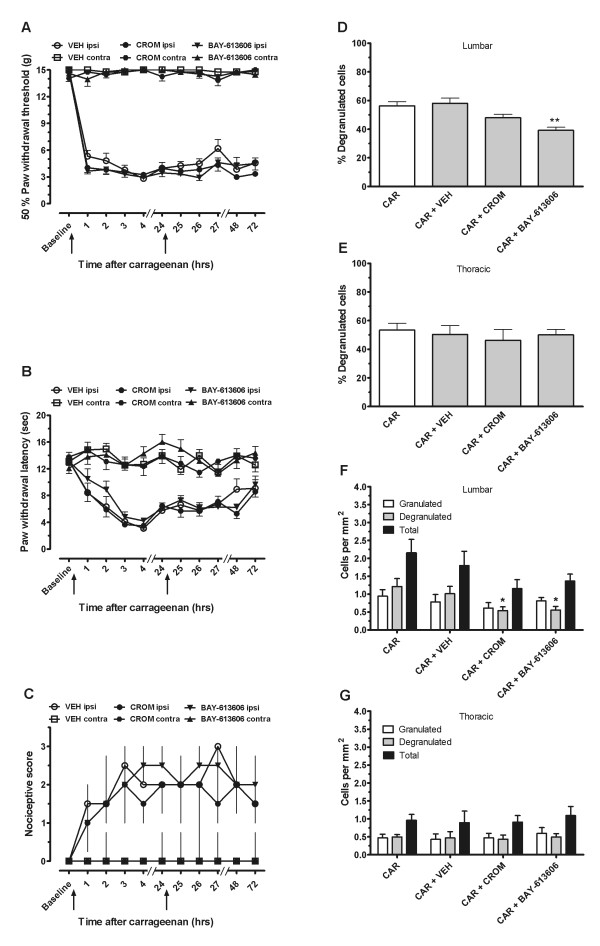
**Effect of intrathecally administered mast cell inhibitors on carrageenan hyperalgesia and mast cell degranulation and density**. Intrathecal administration of either cromolyn or BAY-613606 in female rats before and after carrageenan (at timepoints shown by ↑) does not inhibit mechanical hyperalgesia (A), thermal hyperalgesia (B), or cold allodynia (C). However, BAY-613606 significantly reduces the percent mast cell degranulation in lumbar dura mater (D) (** = P < 0.001) but not in the thoracic dura mater (E) of female rats, while both cromolyn and BAY-613606 inhibit the increased degranulated mast cell density induced by carrageenan in female rats in the lumbar dura mater (F) (* = P < 0.05) but not in the thoracic dura mater (G).

As with capsaicin experiments, the antinociceptive effects of intrathecal administration of the two mast cell inhibitors, cromolyn and BAY-613606, were also examined in female rats. Since the peak mast cell degranulation was found at 24 hours post-carrageenan in the previous experiment, a double injection protocol was used for drug administration, prior to and at approximately 24 hours post carrageenan (after a second "baseline" testing). As compared to intrathecal vehicle, administration of intrathecal cromolyn or BAY-613606 did not inhibit carrageenan-induced mechanical hyperalgesia, either after the first or the second injection (Figure 6A). On the ipsilateral hindpaw, repeated measures two-way ANOVA showed only a significant effect of time (F_10, 210 _= 186.8; P < 0.001), but no significant effect of group or interaction, or any differences on the contralateral hindpaw. For thermal hyperalgesia (Figure 6B), there was also only a significant effect of time (F_10, 210 _= 30.70 P < 0.001), but no significant effect of group or interaction, and no differences on the contralateral hindpaw. As for cold allodynia (Figure 6C), nonparametric repeated measures ANOVA revealed significant difference between groups on the ipsilateral side (Friedman statistic = 6.05 P > 0.05) only. However, one-tailed Mann-Whitney t-tests revealed no significant differences at any timepoints between vehicle treatment and cromolyn or BAY-613606.

In order to show that the administration protocol used in behavioral experiments indeed inhibited mast cells, we collected dura mater 3 hours after the second injection from separate groups of animals treated with the same dosing protocol used in the above behavioral experiments. Comparing percent degranulation in the different groups (Figure 6D), one-way ANOVA revealed a highly significant effect of group (F_3, 27 _= 8.83; P < 0.001). Although there appears to be a reduction with both cromolyn and BAY-613606, post-hoc analysis revealed a significant difference only with BAY-613606 (P < 0.05), and no change with vehicle or cromolyn. In the thoracic dura (Figure 6E), there were no significant differences between groups. Total mast cell density or granulated cell density in the lumbar dura mater was not significantly different between groups. However, there were differences in degranulated cell density in the lumbar dura with a significant reduction by both drug treatments (Figure 6F). One-way ANOVA revealed a significant effect of group (F_3, 27 _= 4.05; P < 0.05) and subsequent post-hoc analysis revealed a significant decrease of degranulated mast cell density with both cromolyn (P < 0.05) and BAY-613606 (P < 0.05), as compared to no drug. There was no effect of the vehicle injections. In the thoracic dura (Figure 6G), there were no significant differences in total, granulated, or degranulated cell densities between carrageenan alone, or with vehicle, cromolyn, or BAY-613606.

## Discussion

Dural mast cells have been mainly investigated in the cerebral dura mater, where density is high and they are close to sensory nerve endings [24]. Substantial evidence suggests that they are involved in migraine pathophysiology [16]. Mast cell degranulation with systemically-administered Compound 48/80 activates meningeal nociceptors, phospho-ERK expression, and spinal trigeminal nucleus cFos activation [15]. Mast cell derived mediators such as serotonin, prostaglandins, and histamine can activate and sensitize meningeal nociceptors [22] and COX-1 is expressed in dural mast cells [25]. It is known that lumbar dural mast cell number varies during development [17], although dural mast cell function remains under speculation. The current study is the first systematic examination of lower spinal dura mast cells in male and female adult rats using two different pain paradigms.

The position of the spinal cord grey matter close to the CSF at the lumbar spinal cord level provided grounds to hypothesize a potential impact of mast cell-derived mediators on superficial dorsal horn neurons. Consistent with this hypothesis, application of supernatant from activated cultured mast cells, particularly from those which were longer activated and secreted newly formed mediators, induced significant mechanical hyperalgesia and LTP at spinal synapses of C-fibres. This suggests a potential contribution of preformed mediators, but a much stronger contribution of newly formed mediators. One hour stimulation of mast cells with TNP is sufficient to induce degranulation, release of histamine and other preformed mediators [26]. Intrathecal histamine administration is pro-nociceptive in mice [27] and spinal histamine receptor antagonists show anti-nociceptive efficacy in various animal models [28]. Given the heterogeneity of mast cells between tissues responsible for their phenotype [1,29] and even within the dura [17], we believe that using mouse bone-marrow cultured mast cells which are less homogeneous than their rat equivalents [30] is still appropriate here. The concentration of cytokines in supernatant was within the same range to that detected by spinal in-vivo microdialysis studies [31] and that of other electrophysiological and behavioral studies. Interestingly, spinally-applied TNFα in the picogram range does not induce *in vivo *spinal cord LTP [32], although intrathecal IL-6 in the nanogram range induces mechanical allodynia [33]. Other mediators such as IL-4 or IL-10 produced by mast cells are not known to be pro-nociceptive [34] and the release of mast cell mediators may also be a homeostatic mechanism [35]. While it is interesting to speculate which specific mediators may be important in inducing the effects seen in our study, it is likely that the combination of preformed and newly formed mediators is physiologically relevant and more important. Persistent peripheral nociceptive stimuli for greater than 24 hours, such as in peripheral hindpaw inflammation, may lead to different combinations of mast cell mediators and potentially to a more important activation of dural mast cells than brief peripheral nociceptive stimuli.

Previous studies have shown that peripheral application of 1% capsaicin induces LTP at spinal synapses of C-fibers [23]. In this study, we found that this dose induces a significantly increased percentage of mast cell degranulation at 3 hours post-application. Since no difference was seen between naïve, vehicle, and 10 minutes post-capsaicin, this suggests that the 3 hour effect is not due to injection stress or the initial nociceptive behaviors induced by capsaicin. This is interesting given that mast cells can degranulate rapidly within minutes [36]. Nevertheless, the intense afferent barrage produced by capsaicin appears to switch granulated mast cells to become degranulated, but not to increase the mast cell number. While mast cell staining with toluidine blue allows for clear visualization of degranulation by staining the granules, it is possible that results may be influenced by loss of mast cell staining with complete degranulation [37], although we did not detect significant decreases in cell numbers after treatments. There was no effect of mast cell inhibition on capsaicin-induced flinching and both cromolyn and BAY-613606 were unable to reduce capsaicin mechanical hyperalgesia, suggesting that this increase is not necessary for mechanical hyperalgesia. Although we were not able to completely inhibit mast cells, the effects of BAY-613606 were stronger than cromolyn reaching below basal levels.

Carrageenan injection produces a peripheral inflammatory stimulus activating afferents from the entire hindpaw that persists longer than capsaicin. Surprisingly, we could find no significant mast cell changes in male rats. However, when we examined female rats, there was a highly significant increase in lumbar dural mast cell density at 24 hours post-carrageenan. This density increase is different from capsaicin and was also seen with 1% carrageenan. Since we did not see a percent increase in mast cell degranulation and no changes to the thoracic region, this could suggest increased localized infiltration or attraction of mast cells. Interestingly, mast cells may also be activated to release mediators without obvious degranulation or in a selective manner [38] and there exist mucosal, connective, and mixed types of mast cells in spinal dura with potentially different functional significance [17]. It may be speculated that mast cells may infiltrate the dura and arrive intact from the peripheral circulation. While in the current study, we did not attempt to localize the source of mast cells or their anatomical position on the dura, we did not find mast cells inside the spinal cord in the 24 hour carrageenan treated females (data not shown). For example, it is known that mast cells may cross blood vessel walls and their mediators may modulate the blood brain barrier [39,40]. Studies of CNS mast cells on blood brain/spinal cord barrier regulation in animal models of pain are lacking.

Mast cells are known to express estrogen receptors [41] and can be activated by estrogens which increase calcium influx and enhance degranulation and mediator release. These effects can be blocked by estrogen receptor antagonists or calcium chelators [42]. The estrous cycle influences thalamic mast cell degranulation [43] and hormones can increase mast cell density in rats [13]. In our study, we did not find any significant changes in thalamic mast cells in rats after carrageenan, although we were unable to conclude about the influence of the estrous cycle. Nevertheless, the brains and dura mater were collected from the same animals, thereby further suggesting that the dural mast cell changes are not widespread. It should also be noted that thalamic mast cells are implicated in multiple functions including sexual reproduction [44], blood brain barrier regulation [40], and stress [45]. Administration of aspirin, but not morphine, decreases rat thalamic mast cell numbers [46]. Nevertheless, we report here an intriguing mast cell sex difference in the spinal dura mater. Various studies have suggested an interaction of hormones and sex with mast cells and potentially pain behaviors. Prevalence of migraine, mast cell diseases, and immediate-hypersensitivity type reactions are particularly prominent in female populations and may relate to hormonal status [47,48]. Changes in estrogen levels may be linked to migraine attacks in women, perhaps due to cerebral mast cell interaction [49]. Future studies may address specifically the role of the estrous cycle on dural mast cells.

We successfully inhibited capsaicin and carrageenan-induced changes in lumbar dural mast cells with two different drugs, although these did not have significant anti-nociceptive effects. Higher intrathecal dosing or volume were not deemed appropriate as they either produced side effects upon injection in the case of cromolyn, or were unable to further reduce mast cell degranulation in the case of BAY-613606 (data not shown). Peripherally, cromolyn has been shown to be anti-nociceptive when administered in the formalin test [7]. Cromolyn, known as a "mast cell stabilizer", has been used in the treatment of asthma and allergic rhinitis [50,51]. It reduces neurogenic inflammation and inhibits mast cell mediator release [52]. Cromolyn has also been suggested to inhibit chloride transport in mast cells, epithelial, and nerve cells [53], and also inhibit substance P binding and adenosine-induced plasma extravasation [54]. The Syk inhibitor BAY-613606 has been shown to be highly selective for the Syk family of tyrosine kinases, which are important signaling molecules involved in mast cell activation and degranulation [55]. When administered orally in rats, it reduces mast cell degranulation, histamine release, and inflammation, more potently than cromolyn [56]. Other Syk inhibitors reduce inflammation in animal models and have been suggested for clinical treatment of rheumatoid arthritis and inflammatory bowel syndrome [57]. Like cromolyn, Syk inhibitors are not fully specific for mast cells since Syk is expressed on a variety of cell types including basophils, leukocytes, platelets, dendritic cells and macrophages [58]. Nevertheless, we found that both cromolyn and BAY-613606 administered intrathecally inhibited increased percentage of mast cell degranulation and degranulated cell density in a localized fashion. Other potentially more powerful methods of mast cell inhibition including the use of transgenic animals such as mast cell-deficient rats [59], or the assessment of different behavioral endpoints will be interesting for use in future studies.

## Conclusion

Our results demonstrate that mast cell mediators may be sufficient to amplify spinal nociception, although lumbar dural mast cells do not appear necessary in capsaicin hyperalgesia and carrageenan inflammatory pain. Our results further demonstrate a sex difference in spinal dural mast cells after carrageenan inflammation. Future studies can test the role of dural mast cells in chronic disease animal models, such as for example back pain [60].

## Methods

### 1. Animals

Animal experiments were approved by the ethic committees of the Medical University of Vienna and the Austrian Ministry for Science and Research (BMWF). These experiments conform to the standards as set out by the European Union and the International Association for the Study of Pain.

Male and female Sprague-Dawley rats (Medical University of Vienna breeding facility; Himberg, Austria), weighing between 175 and 250 grams, were used for all experiments. Animals were kept on a 12/12 hour light/dark cycle and housed 4-6 per cage. Food and water was provided ad libitum. Behavioral experiments were performed between the hours of 10 AM and 6 PM. Animals were habituated to the animal facility for approximately 1 week and were handled by the experimenter at least twice prior to the start of behavioral experiments.

### 2. Drugs and drug administration

Capsaicin 1% or 0.2% (dissolved in 80% saline, 10% ethanol, and 10% Tween 80 with mild heating) and vehicle were administered intradermally in briefly restrained awake animals, using a 30 gauge needle in a 25 μl volume. Carrageenan 3% or 1% (dissolved and vigorously mixed in 100% saline) was administered intradermally using a 30 gauge needle in a volume of 100 μl, while the animal was briefly anesthetized with an inhalable anesthesia mixture of oxygen, isoflurane, and nitrous oxide (N_2 _O). Compound 48/80 (Sigma-Aldrich, Vienna, Austria) was dissolved in saline and administered intraperitoneally using a 25 gauge needle. Sodium cromoglycate (Sigma-Aldrich, Vienna, Austria) was dissolved in saline and BAY-613606 (Sigma-Aldrich, Vienna, Austria) was prepared in distilled water in a 10× stock of 10 mg/ml and then diluted to a final dose of 1 mg/ml in saline for injection. They were both administered intrathecally by lumbar puncture [61] in a 10 μl volume using a 27 gauge Hamilton syringe, under brief isoflurane/N_2 _O anesthesia. Successful injections were confirmed by a characteristic tail flick.

### 3. Mast cell activation and supernatant preparation

Mast cell supernatant was prepared as previously described by [62]. Five-week cultured bone marrow derived mast cells (in RPMI 1640 medium supplemented with 10% heat-inactivated fetal calf serum, 2 mM L-glutamine, antibiotics, 50 μM β-mercaptoethanol, with addition of 5 ng/ml mouse-recombinant interleukin-3 (IL-3) from two 8-10 week-old C57BL/6 mice were used. On day 35 of culture, cells were checked by flow cytometry for the expression of cell surface markers and were at least 95% pure as indicated by c-kit and FcεRI expression. Cells were then seeded at 1 million per ml and primed with immunoglobulin E (1:500) overnight. 24-well plates were coated overnight with phosphate-saline buffer or 2, 4, 6 trinitrophenyl-bovine serum albumin (TNP-BSA) (300 ng/ml) in PBS. Next day, 24-well plates were blocked with 3% detoxified BSA in PBS. Cells were then counted by CasyCounter and seeded at a concentration of 4 million per ml in cell culture medium. Control condition was seeded in a PBS-coated well, and activations were performed in TNP-BSA coated wells for 1 hour and 24 hours. After the activation step, cells were spun down at 1200 rpm for 5 minutes and the supernatant was snap-frozen and kept at -80°C until use in behavioral and electrophysiological experiments. Within the same time period as the other experiments, the supernatant was analyzed in duplicates for cytokines by ELISA for the presence of TNFα, IL-6, and IL-4 (all from BD Bioscience) according to the instructions of the manufacturer.

### 4. Tissue preparation for mast cell staining and counting

For capsaicin experiments, dura samples were collected at 10 minutes, 3 hours, and 24 hours post-capsaicin or vehicle (n = 7 per group). For carrageenan experiments, dura samples from male and female animals were collected at 3 hours, 24 hours, and 72 hours post-injection (n = 7-13 per group). The brain was also collected from most of the carrageenan-treated animals to count mast cells in the thalamus (n = 6-9 per group). As a positive control, in order to observe dural mast cell degranulation, Compound 48/80 or saline vehicle was injected intraperitoneally using a 25 gauge needle at a dose of 2 mg/kg (n = 7 per group). This protocol has been shown to significantly degranulate cranial dural mast cells and activate meningeal nociceptors [15]. To inhibit mast cells in capsaicin experiments (n = 7 per group), 200 μg of cromolyn, 10 μg of BAY-613606 or vehicle (saline) was injected intrathecally in 10 μl immediately prior to capsaicin and dura samples were collected 3 hours post-capsaicin. To inhibit mast cells in carrageenan experiments (n = 7 per group), the same doses as above of cromolyn, BAY-613606, or vehicle were injected intrathecally in 10 μl twice, once immediately prior to carrageenan administration and once 24 hours after carrageenan administration. The dura sample was then collected 3 hours after the second cromolyn injection (27 hours after carrageenan administration).

#### 4.1 Dura Preparation

Animals were deeply anesthetized with ether and transcardially perfused with 25 ml room temperature saline + heparin (10 U/ml) followed by 175 ml of 4% paraformaldehyde (PFA). The entire spinal cord column and skull were dissected and post-fixed in 4% PFA overnight. The vertebral column was then dissected one vertebra at a time and the roots were carefully cut one at a time, in order to remove the spinal cord with an intact dura. The spinal cord was then placed in a petri dish with PBS. The brain was removed from the skull, washed in PBS, and placed in 20% sucrose overnight, and 30% sucrose for 2 additional days, prior to being snap-frozen in isopentane and stored in -80°C until further processing. Under a magnifying lamp, the lumbar and thoracic spinal cord segments were separated and the dura was carefully excised with fine scissors. It was floated on and spread with the help of a cotton tip on gelatin covered slides, which were then allowed to air-dry for at least 2 hours before staining.

#### 4.2 Thalamic preparation

For thalamic sections, frozen brains were cut into 20 μm coronal sections using a cryostat at -25°C. To avoid double-counting of mast cells, alternating sections were collected. The posterior/caudal thalamus was identified with the help of the Paxinos atlas [63] and was localized during cutting using the shape of the hippocampus as a landmark. This area contains significant numbers of mast cells [43] and it includes the lateral thalamic nuclei, the ventral posteromedial nucleus of the thalamus (VPM), and the ventral posterolateral nucleus of thalamus (VPL), which are suggested to be involved in discriminative aspects of pain processing [64]. Between 18 and 20 sections per animal were used for mast cell staining and counting, and were confirmed to be always within the area of the caudal half section of the thalamus.

#### 4.3 Mast cell staining

Slides were stained in 0.2% toluidine blue for the dura and 0.05% toluidine blue for thalamic sections. A 1% stock solution in 70% ethanol was dissolved in 0.5% NaCl solution (pH 2.2-2.3) and the slides were immersed 30 minutes in the above concentrations. They were then washed twice in distilled water, dehydrated in a series of increasing concentrations of ethanol, followed by placement in butyl acetate ester. Samples were coverslipped using Eukitt^® ^mounting medium and were allowed to dry overnight.

#### 4.4 Counting of mast cells

Mast cell counting was always performed in a blinded fashion by an experimenter that was unaware of the sample identity. Criteria for degranulation were agreed upon between the experimenters and included: loss of purple staining, fuzzy appearance, distorted shape, or multiple granules visible in the vicinity of the cell. Inter-rater reliability of mast cell degranulation status was found to be approximately 0.95 in a group of samples. For dura samples and thalamic sections, the entire surface area of the dura or the left and right thalamus were scanned manually using a light microscope at 200× magnification and mast cells were classified as either granulated or degranulated. Pictures of the dura regions used were then created at 12.5× magnification and the area was calculated with the help of the Cell D software (Olympus).

### 5. In vivo electrophysiology

Electrophysiological recordings were performed as described elsewhere [65]. Animals were intubated using a 16 G cannula and mechanically ventilated (Siemens Servoventilator 900C). Isoflurane in two thirds N_2 _O and one third O_2 _was used to induce (4 vol% inspiratory) and maintain (1.5 vol% expiratory) anaesthesia. Surgical level of anaesthesia was verified by stable arterial blood pressure and the absence of paw withdrawal reflexes after pinching the paw. The right femoral vein and artery were cannulated to allow intravenous infusions and to monitor arterial blood pressure. Muscle relaxation was achieved by 2 μgkg^-1 ^h^-1 ^pancuronium bromide intravenously. The left sciatic nerve was dissected free for bipolar electrical stimulation using a silver hook electrode and was covered with warm paraffin oil. Lumbar segments L4 and L5 were exposed by laminectomy and the dura mater was incised and retracted. Two metal clamps were used for fixation of the vertebral column in a stereotactic frame. An agarose pool was then formed around the exposed spinal segments. The spinal segments were covered with artificial cerebrospinal fluid (CSF) which was carefully removed before application of drugs (see below). C-fibre-evoked field potentials were recorded with glass electrodes (2-3 MΩ) in laminae I and II of the spinal cord dorsal horn in response to stimulation of the ipsilateral sciatic nerve at C-fibre strength. Electrodes were driven by a microstepping motor (SM-6, Luigs & Neumann, Germany). Recordings were made with an extracellular amplifier (EXT-02F, NPI Electronic Instruments, Germany) using a band width filter of 0.1 - 1000 Hz. Signals were monitored on a digital oscilloscope (Tektronix TDS 210) and digitized at a sampling rate of 5 kHz by an A/D converter (DigiData 1200 Series Interface). As test stimuli, single pulses (0.5 ms, 25 V, which are suprathreshold for the activation of C-fibres) were delivered to the sciatic nerve every 5 minutes using an electrical stimulator (Iso-Stim 01D, npi electronic, Germany). Stable responses for 1 hour served as control. Control mast cell culture medium, mast cell supernatant from 1-hour TNP stimulated cells, or mast cell supernatant from 24 hour TNP stimulated cells (n = 6 per group) were diluted 1:5 in artificial CSF. 150 μl of the above substances were superfused on the spinal cord by means of a peristaltic pump. Recordings were performed for at least 4 hours. At the end of the experiment animals were decapitated in deep anaesthesia and the spinal cord removed to locate the recording site. Rhodamine B (0.2%) (Sigma-Aldrich, Vienna, Austria) was added to the pipette solution which consisted of (in mM) NaCl 135, KCl 5.4, CaCl_2 _1.8, Hepes 10 and MgCl_2 _1 and pressure was applied to the electrode (300 mbar, 1 min). The spinal cord was then removed, cryofixed and the rhodamine B spot was localized under a fluorescence microscope. All recordings performed showed that the tip of the recording electrode was located in lamina I or II.

### 6. Behavioral assays

Animals were habituated to the testing rooms for 1 week and handled by the experimenters. One day prior to the experiments and on the day of testing, they were habituated to the behavioral apparatus for approximately 30 minutes. Experiments were performed by an experimenter unaware of treatment groups. Animals were randomized into groups and experiments were blocked with an appropriate control group present in every experiment.

#### 6.1 Capsaicin-induced flinching

Nociceptive behaviors after 0.2% capsaicin injections were quantified by placing rats in clear Plexiglas boxes. For a period of 5 minutes post-injection, total flinching, paw elevation, and licking were measured using a stopwatch. A flinch was timed as persisting for approximately one second.

#### 6.2 Thermal hypersensitivity

Thermal thresholds were measured using the plantar Hargreaves Apparatus (Stoelting Europe, Dublin, Ireland). The animals were placed on a heated glass floor (30°C) and the time latency to hindpaw withdrawal (not due to movement) was measured with application of the light source on the region caudal to the foot pads. Three baseline readings were performed and averaged for analysis. For carrageenan experiments, the contralateral paw was tested first followed by the ipsilateral paw.

#### 6.3 Mechanical hypersensitivity

Mechanical thresholds were measured using a set of calibrated Von Frey filaments (Stoelting Europe, Dublin, Ireland) between 0.25 grams and 15 grams in Plexiglas boxes with animals standing on a wire mesh. For capsaicin experiments, the caudal region behind the ipsilateral footpad was stimulated three times using a three- second stimulation in adjacent areas in this same region. For all other experiments, the central region between the footpads was stimulated with one 10 second stimulation. The contralateral paw was tested first followed by the ipsilateral paw. A positive response was counted when a clear hindpaw withdrawal occurred (1 out of 3 in capsaicin experiments) not due to movement of the animal. Ascending hairs were presented in a systematic manner starting with the 2 gram hair. After the first response was obtained, four additional hairs were presented using the up-down method based on [66]. The 50% paw withdrawal threshold was then interpolated using the formula: 50% g threshold = (10^[xf +k*δ*]^)/10,000, where *x*_f _= value (in log units) of the final von Frey hair used; *k *= tabular value (see [66] for pattern of positive/negative responses; and *δ *= mean difference (in log units) between stimuli in each pattern used.

#### 6.4 Cold hypersensitivity

Cold allodynia testing was performed using the acetone test. Using a pipette, 50 μl of acetone were applied to the plantar hindpaw center while the animal was standing on a wire mesh. The animal was observed for 60 seconds and a nociceptive score based on a modified method described in [67] with the following 4 point scale: 0-no response, 1-brief flicking less than 3 seconds, 2-flicking and other behaviors such as paw elevation or licking for more than 3 seconds, 3-prolonged flicking and other behaviors such as paw elevation and licking for more than 10 seconds).

### 7. Behavioral experimental design

#### 7.1 Effect of mast cell supernatant on nociception

Supernatant from non-activated mast cells, supernatant from mast cells activated for 1 hour or 24 hours or cell culture medium was injected undiluted intrathecally in male Sprague-Dawley rats (n = 6 per group). Mechanical hyperalgesia, thermal hyperalgesia, and cold allodynia was measured at 30 minutes, 2 hours, 4 hours, 24 hours, 48 hours, 4 days, and 1 week post injection.

#### 7.2 Capsaicin nociception and mast cell inhibition

To assess the effect of mast cell inhibition on capsaicin mechanical hyperalgesia, male animals (n = 8 per group) were injected intrathecally with cromolyn immediately prior to intradermal 1% capsaicin. Mechanical hyperalgesia was measured on the ipsilateral hindpaw at 30 minutes, 1 hour, 1.5 hours, 2 hours, 3 hours, 4 hours, and 24 hours post-capsaicin. The effect of mast cell inhibition with intrathecal cromolyn and BAY-613606 pretreatment (n = 8 per group) was assessed on 0.2% capsaicin-induced flinching behaviors and mechanical hyperalgesia. Flinching behaviors were assessed for the first 5 minutes after capsaicin, while mechanical hyperalgesia was measured starting at 30 minutes, 1 hour, 1.5 hours, 2 hours, 3 hours, 4 hours, and 24 hours post-capsaicin.

#### 7.3 Carrageenan nociception and mast cell inhibition

To assess the effect of mast cell inhibition on carrageenan mechanical hyperalgesia, thermal hyperalgesia, and cold allodynia, female animals (n = 8 per group) were injected intrathecally with 200 μg cromolyn or 10 μg BAY-613606 in 10 μl immediately prior to carrageenan administration and 24 hours after carrageenan administration. Thermal hyperalgesia, mechanical hyperalgesia, and cold allodynia were measured on both the ipsilateral and the contralateral hindpaws starting at baseline (before carrageenan) 1 hour, 2 hours, 3 hours, 4 hours, approximately 24 hours (2^nd ^baseline prior to drug injection), 25 hours (1 hour post second drug injection), 26 hours (2 hours post-second drug injection), 27 hours (3 hours post-second drug injection), 48 hours (24 hours post-second drug injection), and 72 hours post-carrageenan (48 hours post-second drug injection).

### 8. Statistical analyses

All data are expressed as means ± standard error, except for those of the acetone test which are median ± interquartile range. For the analyses of C-fibre evoked field potential areas, mechanical withdrawal thresholds, and thermal latencies, parametric two-way repeated ANOVAs were used followed by Dunnett's Test with vehicle as a control. Electrophysiology data was normalized to the baseline and 30 minute intervals were averaged together. For analysis of cold allodynia, non-parametric Friedman test was performed followed by individual timepoint comparisons to vehicle using one-tailed non-parametric Mann-Whitney U-tests. For mast cell counts, two-tailed t-tests were used to compare vehicle to Compound 48/80. One-way independent measures ANOVA followed by Dunnett's post hoc test was used to analyze percent degranulation, total mast cell density, granulated mast cell density, and degranulated mast cell density.

## List of abbreviations

ANOVA: analysis of variance; BSA: bovine serum albumin; CNS: central nervous system; COX: cyclooxygenase; CSF: cerebrospinal fluid; IL: interleukin; IgE: immunoglobulin E; LTP: long term potentiation; N_2 _O: nitrous oxide; PBS: phosphate buffered saline; Syk: spleen tyrosine inhibitor; TNF: tumor necrosis factor; TNP: trinitrophenol.

## Competing interests

The authors declare that they have no competing interests.

## Authors' contributions

DX carried out tissue processing, mast cell counting, behavioral assays, data analysis, designed and coordinated all the studies, and drafted the manuscript. SG carried out tissue processing, mast cell counting, behavioral assays, and helped with data analysis and manuscript revisions. RD carried out in vivo electrophysiological studies and helped with their design. EN carried out tissue processing, mast cell counting and helped with manuscript revisions. AA carried out mast cell culture and activation, cytokine assay, and helped with design of mast cell supernatant studies. WE helped with design of mast cell supernatant studies. JS participated in the design of the project and all the studies and in manuscript revisions. All authors read and approved the manuscript.
